# Membrane Stress Caused by Unprocessed Outer Membrane Lipoprotein Intermediate Pro-Lpp Affects DnaA and Fis-Dependent Growth

**DOI:** 10.3389/fmicb.2021.677812

**Published:** 2021-06-07

**Authors:** Digvijay Patil, Dan Xun, Markus Schueritz, Shivani Bansal, Amrita Cheema, Elliott Crooke, Rahul Saxena

**Affiliations:** ^1^Department of Biochemistry and Molecular and Cellular Biology, Georgetown University Medical Center, Washington, DC, United States; ^2^Lombardi Comprehensive Cancer Center, Georgetown University Medical Center, Washington DC, United States

**Keywords:** *Escherichia coli*, DNA replication, acidic phospholipids, lipoprotein biogenesis, DnaA, Fis, MicL-S

## Abstract

In *Escherichia coli*, repression of phosphatidylglycerol synthase A gene (*pgsA*) lowers the levels of membrane acidic phospholipids, particularly phosphatidylglycerol (PG), causing growth-arrested phenotype. The interrupted synthesis of PG is known to be associated with concomitant reduction of chromosomal content and cell mass, in addition to accumulation of unprocessed outer membrane lipoprotein intermediate, pro-Lpp, at the inner membrane. However, whether a linkage exists between the two altered-membrane outcomes remains unknown. Previously, it has been shown that *pgsA*^+^ cells overexpressing mutant Lpp(C21G) protein have growth defects similar to those caused by the unprocessed pro-Lpp intermediate in cells lacking PG. Here, we found that the ectopic expression of DnaA(L366K) or deletion of *fis* (encoding Factor for Inversion Stimulation) permits growth of cells that otherwise would be arrested for growth due to accumulated Lpp(C21G). The DnaA(L366K)-mediated restoration of growth occurs by reduced expression of Lpp(C21G) *via* a σ^*E*^-dependent small-regulatory RNA (sRNA), MicL-S. In contrast, restoration of growth *via fis* deletion is only partially dependent on the MicL-S pathway; deletion of *fis* also rescues Lpp(C21G) growth arrest in cells lacking physiological levels of PG and cardiolipin (CL), independently of MicL-S. Our results suggest a close link between the physiological state of the bacterial cell membrane and DnaA- and Fis-dependent growth.

## Introduction

In *Escherichia coli*, DnaA protein initiates chromosomal DNA replication once, and only once, per cell cycle (Bramhill and Kornberg, 1988; Marszalek and Kaguni, 1994; Baker and Bell, 1998). DnaA shows a high affinity for both ATP (*K*_*D*_ = 0.03 μM) and ADP (*K*_*D*_ = 0.1 μM) (Sekimizu et al., 1987). Early biochemical studies established the role of acidic phospholipids in the *in vitro* rejuvenation of inactive ADP-DnaA to active ATP-DnaA (Sekimizu and Kornberg, 1988; Yung and Kornberg, 1988; Castuma et al., 1993). Limited proteolysis of DnaA in the presence of acidic phospholipids to generate functional fragments revealed a specific region of DnaA involved in membrane interaction (Garner and Crooke, 1996). Independently, the same region was found to insert into fluid acidic phospholipid bilayers when probed with a photoactivatable lipid analog (Garner et al., 1998). Cytolocalization studies involving an allelic replacement of the chromosomal copy of *dnaA* with a gene encoding GFP-DnaA demonstrated a discrete, longitudinal helical arrangement of GFP-DnaA along the cell periphery (Boeneman et al., 2009), supporting that DnaA is located along the inner leaflet of the cytoplasmic membrane.

Genetic studies on acidic phospholipid synthesis revealed that the absence of a functional gene for phosphatidylglycerol phosphate synthase, *pgsA* (Heacock and Dowhan, 1987; Xia and Dowhan, 1995), results in arrested growth due to reduced levels of phosphatidylglycerol (PG) and cardiolipin (CL). Site-directed mutagenesis of the membrane-binding region of DnaA offered a novel perspective on membrane–DnaA initiator protein interactions: a single-point mutation in DnaA at the hydrophobic face of the amphipathic helix, DnaA(L366K), allows growth in cells with depleted acidic phospholipid content (Zheng et al., 2001). DnaA(L366K) cannot serve as the only source of DnaA to initiate DNA synthesis both *in vivo* (Zheng et al., 2001) and *in vitro* (Saxena et al., 2011) and requires limiting levels of wild-type DnaA to function. Biochemical characterization of DnaA(L366K) demonstrated that it behaves like DnaA with respect to nucleotide-binding affinities, ATP hydrolysis, and specificity for PG and CL in promoting nucleotide exchange (Li et al., 2005). *In vitro* DMS-footprinting demonstrated that DnaA(L366K) fails to saturate low-affinity recognition sites I2 and I3 irrespective of its adenine nucleotide-bound state. However, in the presence of a limiting amount of wild-type DnaA, DnaA(L366K) promotes the formation of replication-competent DnaA–*oriC* complexes (Saxena et al., 2011). Moreover, it has also been shown that membrane-mediated DnaA rejuvenation is strongly cooperative with respect to DnaA membrane occupancy. *In vitro*, when DnaA remains present on dioleolylPG liposomes, it could transit between two occupancy states: high-density occupancy (state I) and low-density occupancy (state II) states (Aranovich et al., 2006, 2015). Only low-density occupancy state II is efficient in facilitating the nucleotide exchange that is presumably promoted by macromolecular crowding (Aranovich et al., 2006, 2015). DnaA(L366K) and is shown to require relatively lower amounts of membrane acidic phospholipids to facilitate nucleotide exchange (Aranovich et al., 2007).

In addition to DnaA–acidic membrane association, PG is also required for the biogenesis of outer membrane lipoprotein, Lpp (also known as Braun’s lipoprotein), which is the most abundant protein in the *E. coli* cell envelope (Inouye, 1979). The maturation of pro-lipoprotein (pro-Lpp) involves a series of enzyme-catalyzed reactions starting with lipoprotein diacylglyceryl transferase (Lgt), which catalyzes the transfer of diacylglycerol (DAG) from PG to cysteine 21 of pro-Lpp (Braun, 1975; Sankaran and Wu, 1994; Mao et al., 2016). Signal peptidase II (Lsp) recognizes the diacylglycerated-cysteine and cleaves the signal sequence from pro-Lpp to form apolipoprotein (Paetzel et al., 2002). Previous work has indicated the requirement for equimolar levels of PG to Lpp in the maturation process of pro-Lpp (Suzuki et al., 2002).

In sum, existing findings suggest that inhibited growth of *E. coli* cells lacking membrane acidic phospholipids arises from either defective DnaA-mediated initiation of replication in the vicinity of the cytoplasmic membrane, faulty maturation of major outer membrane lipoprotein, or the intersection of both processes (Inouye et al., 1983; Cronan, 2003; Saxena et al., 2013). However, with respect to the *latter*, the manner by which immature pro-Lpp might affect normal, DnaA-dependent normal growth is still unclear.

The recent understanding of outer membrane homeostasis highlights RNA polymerase sigma factor RpoE (σ^24^/σ^*E*^)-dependent, MicL-S small RNA-mediated regulation of endogenous Lpp levels for cell survival at the early stationary phase (Guo et al., 2014; Nicoloff et al., 2017). The extracytoplasmic function (ECF) sigma factor σ^*E*^ responds to outer membrane disorders in a complex signal cascade involving degradation of the inner membrane anti-sigma factor, RseA, and subsequent release of σ^*E*^ into the cytoplasm (Raivio and Silhavy, 2001; Chaba et al., 2007; Lima et al., 2013; Klein and Raina, 2019). Active σ^*E*^ transcribes multiple small RNAs, like MicA, RybB, and MicL to translationally inhibit outer membrane porins and lipoproteins (Johansen et al., 2006; Udekwu and Wagner, 2007; Guo et al., 2014). MicL-S is an 80-bp small RNA derived from MicL (308 bp), present at the 3′UTR adjacent to the gene-coding region for copper homeostasis protein (*cutC*), and targets specifically *lpp* mRNA (Guo et al., 2014). Furthermore, the capacity of a suppressor mutation in *lpp* (*lpp-2*) or deletion of *lpp* to restore growth to *pgsA-*null cells with a lower acidic phospholipid content supports the requisite for PG to allow maturation of pro-Lpp (Miyazaki et al., 1985; Kikuchi et al., 2000). Related, inhibited growth caused by expression of Lpp(C21G) substantiates the need for the thioester diacylglycerol modification required for proper Lpp maturation (Inouye et al., 1983).

The bacterial nucleoid resides in the vicinity of the cytoplasmic side of the inner membrane, which also serves as an important site for Lpp biogenesis. The nucleoid is dynamic and flexible in nature and undergoes various changes to regulate complex processes, including DNA replication, DNA recombination, and gene expression (Dorman and Deighan, 2003; Luijsterburg et al., 2006; Dillon and Dorman, 2010). The organization and structure of the bacterial nucleoid are affected by small nucleoid-associated proteins (NAPs), which bind to chromosomal DNA (R.T Dame, 2005; Dillon and Dorman, 2010). NAPs include proteins such as histone-like protein HupA, HupB (functions in DNA topology management) (Varshavsky et al., 1977), H-NS protein (reinforces negative supercoiling to facilitate DNA–protein–DNA bridges) (Dorman et al., 1999; S. Rimsky, 2004), and DNA bending–binding protein such as integration host factor IhfA, IhfB (S Khrapunov et al., 2006), and Fis (Factor for Inversion Stimulation) (Gille et al., 1991). Particularly, Fis protein, which is abundantly present during exponential growth, binds to numerous sites on the chromosome and acts as a global transcription factor (Cho et al., 2008). *In vitro* studies indicated that Fis also binds to specific chromosomal sites present in *oriC* (Wold et al., 1996) and *DARSs* (DnaA-reactivating sequences) (Kasho et al., 2014); the latter appears to play a role in rejuvenation of ADP-DnaA to ATP-DnaA (Fujimitsu et al., 2009). Fis and IHF proteins complex with these regions and act as a pleiotropic regulator of initiation of replication to inhibit untimely initiations (Wold et al., 1996; Kasho et al., 2014), probably *via* altering of the DNA supercoiling (Margulies and Kaguni, 1998). *In vivo*, *E. coli* lacking *fis* are able to grow when cultured in minimal media; however, when cultured in rich media, the rapidly growing cells had increased mass but possessed fewer origins per cell, having initiated replication in an asynchronous manner (Flatten and Skarstad, 2013).

Although reports indicate that NAPs are needed to regulate several important housekeeping genes, whether they serve in a manner to link two membrane-associated outcomes in acidic phospholipid-deficient cells, perturbed chromosomal replication and defective biogenesis of outer membrane lipoproteins, has not been addressed. Here, we investigate whether accumulated Lpp(C21G) intermediates poison DnaA-dependent *oriC*-mediated replication initiation in *E. coli* with normal levels of acidic phospholipids, and if any auxiliary initiation factors play a role in linking the two membrane-associated processes. We also examine the possible role of the σ^*E*^-MicL/Lpp protective loop in the ability of DnaA(L366K) to facilitate growth of cells carrying mutant Lpp(C21G) or lacking the ability to synthesize the acidic phospholipids.

## Materials and Methods

Restriction enzymes were purchased from New England Biolabs (NEB). All polymerase chain reactions, unless mentioned otherwise, were performed using Q5 High fidelity DNA polymerase (NEB). PCR primers were designed using GeneRunner program Version 6.5.52^1^ Primers used in the study were custom synthesized from Integrated DNA Technologies. Various ingredients to reconstitute minimal media (M9) or Luria broth (LB) were purchased from Sigma or VWR.

### Media, Bacterial Strain, and Plasmids

Minimal media supplemented with 0.4% glucose and 0.5% Casamino acids were used to grow cells both in liquid culture and in agar plates. Supplementary Table 1 showed *E. coli* strains and the plasmids used in the study. *E. coli* strain BW25113 (*lpp*^+^) or JW1667-5 (*lpp*^–^) was obtained from *E. coli* genetic stock center^2^ and their different derivatives (used in this study) were constructed in laboratory using plasmid-inducible lambda-red recombinase-mediated genome modification system.

### Plasmid Construction

Plasmid pSC were generated by replacing pBR322 origin of replication and the ampicillin-resistance gene from plasmid pBAD24c (Guzman et al., 1995) with p15A origin of replication and tetracycline-resistant gene from the pACYC184 vector (Chang and Cohen, 1978; Rose, 1988). The *dnaA* or *dnaAL366K* allele was removed from previously described plasmids, pZL606 and pZL607 (Zheng et al., 2001), by digesting with *Nde*I and *Sty*I, and inserting into the same restriction enzyme site in pSC plasmid. An open reading frame for *fis* gene was amplified by PCR using chromosomal DNA as a template and a pair of primers (Supplementary Table 2) carrying *Nde*I and *Sty*I. The amplified product was inserted into the corresponding sites of plasmid pSC to place *fis* allele under arabinose-inducible promoter. Similarly, plasmid pC2 (Nakamura and Inouye, 1982, we received as a gift from Dr. Thomas Silhavy) carries *lpp*(C21G) gene cloned between *Xba*I and *EcoR*I, and placed under control of an IPTG-inducible promoter. Wild-type *lpp* or *lpp*(ΔK58) was amplified by PCR using chromosomal DNA as a template and a pair of primers containing *Xba*I and *EcoR*I restriction sites. The amplified fragments were inserted into the corresponding sites of the pC2 plasmids. The transformants were screened to select for the integration of the desired products by restriction digestion analysis, which were later confirmed by the automated Sanger DNA sequencing method (Genewiz).

### Lambda-Red [λ-Red] Recombinase-Mediated Genetic Recombineering

For insertional-deletion of target genes, the relevant bacterial strains were electroporated with one of the two pKD46 (Datsenko and Wanner, 2000; Baba et al., 2006) derivatives carrying λ-red recombinase genes (*exo*, *beta*, and *gam*): pKD-sg-ack (Reisch and Prather, 2015) or pSIJ8 (Jensen et al., 2015) for the insertion of antibiotic resistance gene cassette. For the homology-directed recombination (HDR), donor DNA was designed to carry (1) 50–60 base-pair-long 5′-upstream and 3′-downstream DNA sequence of the gene to be deleted, and (2) reverse DNA sequence of the antibiotic resistance gene cassette to maintain expression of the antibiotic resistance gene through its promoter. All donor DNA for HDR with desired antibiotic cassette were amplified using plasmid pCas9cr4 (Reisch and Prather, 2015), for which primers were designed based on its gene sequence.

### Cell Viability Assay

*E. coli* cultures were prepared by growing cells overnight (12–14 h) in M9+CAA+Glu media supplemented with appropriate antibiotics (ampicillin: 100 μg/ml, tetracycline: 12.5 μg/ml, and kanamycin: 50 μg/ml) at 37°C. Overnight grown cultures were diluted to inoculate an equal number of cells (∼10,000 cfu/ml) in fresh M9+CAA+Glu media supplemented with appropriate antibiotics in the presence of inducers. To perform growth curve analysis, optical densities (OD at 600 nm) were recorded after every 60 min (±10) for at least 12–18 h. Additionally, samples (1 ml) were drawn at different time point intervals from separate conditions to analyze cell viability and level of protein expression. For viability assays, OD (600 nm) values for cell suspensions were normalized to 0.6 × 10^9^ cells/ml, which were then serially diluted by 10-fold dilutions up to 10^–5^. A fixed volume (25 μl) of each serial dilution was plated on M9+CAA+Glu agar plates to obtain 200–500 colonies/plates. Viability was also assessed using a spotting assay in which serially diluted cell suspensions (2.5 μl) grown in the absence of inducer were spotted on M9+CAA+Glu-agar plates carrying various concentrations of isopropyl-β-D-galactopyranoside (IPTG), or L-Arabinose (Sigma), or both. One-way analysis of variance (ANOVA) with multiple comparison tests was performed to compare viability among groups using Prism 8 (GraphPad).

### Immunoblotting

Based on the OD (600 nm), equal numbers of cells were pelleted by centrifugation at 16,000 × *g* for 10 min at 4°C. Cell pellets were resuspended in 30 μl of PBS and 12 μl (4×) of SDS sample buffer and subsequently boiled for 5 min before resolving proteins with 19% SDS-polyacrylamide-urea gels. Proteins were transferred to PVDF membranes and visualized by Ponceau S staining to evaluate the total protein content. Expressions of Lpp and Lpp(C21G) proteins were detected using anti-Lpp antiserum, and the expression of DnaA(L366K) was detected using the anti-DnaA antiserum. Subsequently, immunoblots were treated with secondary stabilized peroxidase-conjugated goat anti-rabbit antibody (Thermo Fisher Scientific) and processed with SuperSignal West Femto Maximum Sensitivity Substrate (Thermo Fisher Scientific) using AI600 Imager (GE Healthcare Biosciences). Experiments were performed with at least three biological replicates. ImageJ (Version 1.51) software was used to quantitate the band intensity of proteins detected by immunoblots and Ponceau S staining.

### Lipidomic Analysis

For lipidomic analysis, MDL12 and MDL12Δ*fis* cells were grown to obtain 10–50 million cells/ml (cell number was determined by dilution plating). Bacterial cells were collected (13,500 rpm at 4°C for 10 min), and the cell pellets were flash-frozen and stored at −80°C until further analyzed. Frozen cell pellets were thawed on ice and resuspended in 40 μl of chilled water/methanol/IPA [35:25:40]. The mixture was subjected to freezing (plunging the tube containing mixture into dry ice for 30 s) and rapid heating (plunging tubes into a 37°C water bath for 90 s) twice before sonication at 30 kHz for 30 s mixed with 100 μl of ice-chilled isopropyl alcohol containing internal standards. Subsequently, the samples were placed on ice for 30 min and transferred to −20°C for 30 min following centrifugation at 13,500 rpm at 4°C for 20 min. Supernatants were collected to perform mass spectrometric (MS) analysis using the targeted LCMS-MS approach where they were resolved on an Xbridge Amide 3.5 μm, 4.6 × 100 mm column (Phenomenex) online with a triple quadrupole mass spectrometer (5500 QTRAP, SCIEX, United States) operating in the multiple reaction monitoring (MRM) mode. The declustering potential, collision energies, cell exit potential, and entrance potential optimized for each metabolite to obtain maximum ion intensity for parent and daughter ions *via* manual tuning in Analyst 1.6.3 software (SCIEX, United States). Signal intensities from all MRM Q1/Q3 ion pairs for the analyte were ranked to ensure the selection of the most intense precursor and fragment ion pair for MRM-based quantitation. This approach resulted in the selection of declustering potential, collision energies, cell exit potential, and entrance potential that maximized the generation of each fragment ion species. The metabolite ratios were calculated by normalizing the peak area of endogenous metabolites within samples normalized to the internal standard for every class of lipid molecule. The sample queue was randomized, and solvent blanks were injected to assess sample carryover. Pooled quality control (pooled QC) samples were injected after every eight samples to check for instrumental variation. Like pooled QC samples, Sciex standard QC plasma samples were also injected for lipidomic data analysis. For CL data acquisition, bovine heart extract was used as quality control. Data normalized to QC variance. QC normalized data and imputed MRM data were processed using MultiQuant 3.0.3 (Sciex). The relative quantification values of analytes were determined by calculating the ratio of peak areas of transitions of samples normalized to the peak area of the internal standard specific for every class. All statistical analyses were performed using Prism 8 (GraphPad). Two or more groups were compared with one-way ANOVA, and data for selective 124 lipid metabolites were represented in the heatmap. Multiple *t*-tests were performed using log-transformed values of metabolite peak ratio and compared using volcano plot. For volcano plots, the *p*-value cutoff was set at *p* = 0.05, and log2 fold change (fold difference) that corresponds to the statistical significance was set at ± 2.

## Results

### Induction of Lpp(C21G) Expression Inhibits Growth That Is Restored by Ectopic Expression of DnaA(L366K)

*E. coli* cells lacking acidic membranes, particularly PG, caused growth-arrested phenotype. Insufficient levels of PG interrupt two important cellular processes, (i) DnaA-mediated process of replication initiation at the chromosomal origins (Fingland et al., 2012), and (ii) the maturation and proper sorting of outer membrane lipoprotein, Lpp (Matsuyama et al., 1995). Overexpression of a mutant DnaA, DnaA(L366K) protein, suppresses the growth defect in cells unable to synthesize PG. A previous study (Inouye et al., 1983) had demonstrated that bacterial cells carrying cellular levels of PG but overproducing mutant outer membrane lipoprotein, Lpp(C21G), which is unable to be processed by PG, also resulted in growth-arrested phenotype. Wondering whether there is a link of some fashion between the two growth-arrest phenotypes led us to assess the capacity of DnaA(L366K) to restore growth in bacterial cells exogenously overproducing Lpp(C21G). For this, we developed a two-plasmid system for the simultaneous expression of the Lpp(C21G) and DnaA(L366K) (see “Materials and Methods”). The genes for Lpp and Lpp(C21G) were cloned under dual Lpp and IPTG-inducible LacUV5 promoters (Inouye et al., 1983; Supplementary Table 1). Lpp or Lpp(C21G) was expressed either *via* the constitutive *lpp* promoter in the absence of IPTG or *via* the combined *lpp* and *lac* promoters in the presence of IPTG in a *lacI*^+^ (Nakamura and Inouye, 1982; Supplementary Table 1) genetic background. The genes for DnaA and DnaA(L366K) were cloned under control of the L-arabinose-inducible pBAD promoter.

**FIGURE 1:**
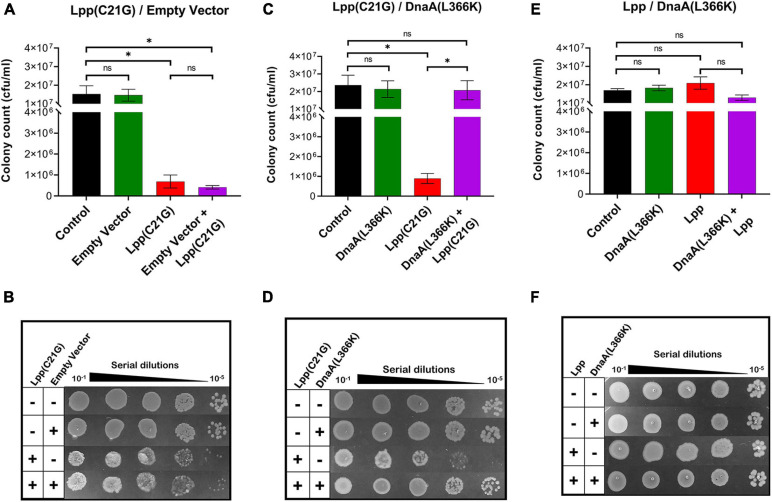
DnaA(L366K) overexpression rescues growth in cells expressing Lpp(C21G). **(A)** Viability of *lpp-null* cells transformed with empty vector, and plasmids expressing Lpp(C21G). No inducer control (black), in the presence of 0.2% Arabinose (green) for empty vector, 50 μM IPTG (red) for Lpp(C21G), and 50 μM IPTG + 0.2% Arabinose (purple) for both Lpp(C21G) and empty vector expression in M9+CAA+Glu media. **(B)** Serial dilutions of *lpp-null* cells, capable of expressing plasmid-derived Lpp(C21G) and empty vector, on agar plates. **(C)** Viability of *lpp-null* cells expressing DnaA(L366K) and Lpp(C21G). No inducer control (black), in the presence of 0.2% Arabinose (green) for DnaA(L366K), 50 μM IPTG (red) for Lpp(C21G), and 50 μM IPTG + 0.2% Arabinose (purple) for both Lpp(C21G) and DnaA(L366K) expression in M9+CAA+Glu media. **(D)** Serial dilutions of *lpp-null* cells, capable of expressing plasmid-derived Lpp(C21G) and DnaA(L366K), on agar plates. **(E)** Viability of *lpp-null* cells expressing DnaA(L366K) and Lpp. No inducer control (black), in the presence of 0.2% Arabinose (green) for DnaA(L366K), 50 μM IPTG (red) for Lpp, and 50 μM IPTG + 0.2% Arabinose (purple) for both Lpp and DnaA(L366K) expression in M9+CAA+Glu media. **(F)** Serial dilutions of *lpp-null* cells, capable of expressing plasmid-derived Lpp and DnaA(L366K), on agar plates. Viability is expressed in cfu/ml and presented on a linear scale. Data are means ± SEM of at least three independent experiments. ^∗^*p* < 0.05, ns *p* > 0.05 in one-way ANOVA with Dunnett’s multiple comparison test.

As reported before, we confirmed that *E. coli lpp-null* cells carrying plasmid-borne mutant *lpp*(C21G) grown in the presence of inducer (as low as 50 μM IPTG) resulted in a 27.4-fold reduction (*p* < 0.001) in cell viability (Supplementary Figures 1C,D). The bacterial cells overproducing Lpp(C21G) when transformed with plasmid vector pSC in the absence or presence of inducer (0.2% arabinose) did not show any changes in the restricted growth (Figures 1A,B). The ectopic expression of DnaA(L366K), however, restored growth, with cells retaining viability (*p* < 0.04, Figures 1C,D). In contrast, overexpression of DnaA(WT) either alone, as reported elsewhere (Atlung et al., 1987; Pierucci et al., 1989; Flatten et al., 2015), or in conjunction with Lpp(C21G) led to poor growth (Supplementary Figures 2A,B). As a control, similar overexpression of DnaA(L366K) in cells expressing Lpp(WT) had no effect (Figures 1E,F).

**FIGURE 2:**
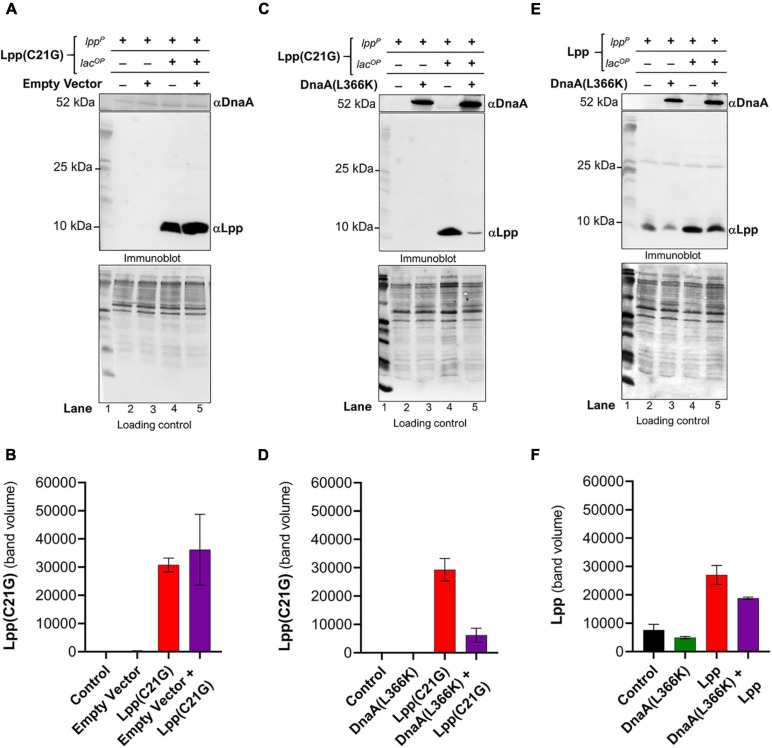
*Ecto*pic expression of DnaA(L36*6K*) reduces levels of Lpp(C2*1G*) even when the inducer is present. **(A,B)** Immunoblotting for the detection of DnaA and Lpp(C21G) and quantitative analysis of Lpp(C21G) levels. **(C,D)** Immunoblotting for the detection of DnaA(L366K) and Lpp(C21G) and quantitative analysis of Lpp(C21G) levels. **(E,F)** Immunoblotting for the detection of DnaA(L366K) and Lpp and quantitative analysis of Lpp levels. Lpp(C21G) and Lpp are expressed via Lpp (lpp^*P*^) and Lac (lacUV5^*OP*^) promoters. Loading controls for immunoblotting are ponceau-S staining for total protein normalization presented in grayscale. Data are means ± SEM of at least three independent experiments.

Given that DnaA(L366K) restored growth when the inducer for Lpp(C21G) was present, we next questioned whether DnaA(L366K) affects the cellular levels of Lpp(C21G). Immunoblots revealed that pSC vector alone did not affect the expression of Lpp(C21G) (lanes 4–5, Figures 2A,B); however, overproduction of DnaA(L366K) caused an approximate sevenfold reduction in levels of Lpp(C21G) (lane 5, Figures 2C,D), as compared to cells expressing Lpp(C21G) alone (lane 4, Figure 2C). When compared to the reduction of Lpp(C21G) levels, we observed significantly less reduction in Lpp arising from overexpression of DnaA(L366K) (lanes 4–5, Figures 2E,F), suggesting a *lpp* mutation-specific response to DnaA(L366K).

Separately, we confirmed the differences in exogenous expression of Lpp and Lpp(C21G) in *lpp-null* cells (Supplementary Figures 1G,H). We observed approximately threefold higher Lpp(C21G) levels (lane 3, Supplementary Figure 1H) when compared with Lpp (lane 3, Supplementary Figure 1G) in the presence of 50 μM IPTG inducer in a manner similar to the expression of Lpp(C21G) (lane 4, Figure 2C; and lane 4, Figure 2E). At the same time, the Lpp(C21G) levels (lane 3, Supplementary Figure 1H) were still observed to be approximately 50-fold less than the endogenous levels of Lpp in *lpp*^+^ cells (lane 6, Supplementary Figure 1G). These results further confirm that inhibited growth in *lpp-null* cells resulted from the accumulation of unprocessed Lpp(C21G), but not Lpp, as shown elsewhere (Inouye et al., 1983).

### Absence of the Promoter Region for MicL-S Small RNA Prevents the Ability of DnaA(L366K) to Decrease Lpp(C21G) Expression and Only Partially Restore Growth

The *E. coli* transcription factor σ^*E*^ has been recognized as an envelope stress-responsive sigma factor that senses an abnormality of the outer membrane integrity. It has been previously reported that abundance of Lpp results in the σ^*E*^ activity and that MicL and Lpp comprise a new regulatory loop that opposes membrane stress (Guo et al., 2014). Interestingly, when cells were grown without IPTG, we only observed expression *via* the *lpp* promoter of Lpp (lanes 2–3, Figures 2E,F) but not Lpp(C21G) (lanes 2–3, Figures 2A,D). This observation indicated the possible role of σ^*E*^-dependent MicL-S-mediated envelope protective mechanisms in preventing expression of Lpp(C21G) from the *lpp* promoter in cells harboring the empty vector (lanes 2–3, Figure 2A) or expressing DnaA(L366K) (lanes 2–3, Figure 2C). However, when cells were treated with IPTG to induce Lpp(C21G), the ectopic expression of DnaA(L366K) was required to decrease Lpp(C21G) levels (lane 5, Figure 2C) and allow growth (Figure 1C), as opposed to the empty pSC vector (lane 5, Figures 1A, 2A). The ability of DnaA(L366K) to reduce levels of Lpp(C21G) led us to question whether the ectopic expression of DnaA(L366K) translationally inhibits Lpp(C21G) *via* early activation of the σ^*E*^-dependent MicL-S-mediated envelope protective loop. To test this, we chose an *E. coli* strain lacking the chromosome region for *cutC* (*cutC-null*, Supplementary Table 1); *cutC* encodes for a copper homeostasis protein. The region for *cutC* comprises an σ^*E*^ binding site for the transcription of MicL small RNA (Guo et al., 2014; Nicoloff et al., 2017). Therefore, the lack of the *cutC* disrupts the σ^*E*^-dependent envelope protective loop by preventing transcription of the MicL-S sRNA. Studies suggest that lack of *cutC* leads to increased levels of endogenous Lpp and affects membrane integrity (Guo et al., 2014). So, for this study, we deleted the gene-encoding region for *lpp* in cells already lacking *cutC* (Δ*cutC*) to test the effect of Lpp(C21G) on cell growth.

**FIGURE 3:**
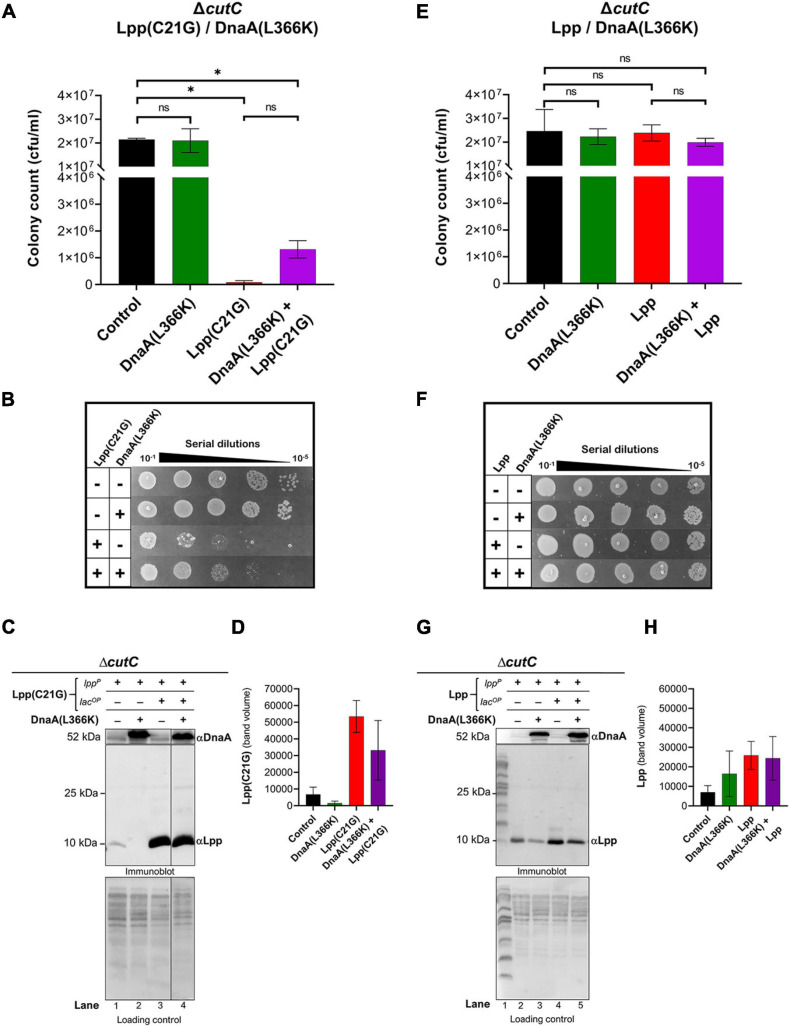
Overexpression DnaA(L366K) res*to*res limited growth but fails *to* reduce levels of Lpp(C2*1G*) when the promoter for MicL-S small RNA is absent. **(A)** Viability of cutC-null cells expressing DnaA(L366K) and Lpp(C21G). No inducer control (black), in the presence of 0.2% Arabinose (green) for DnaA(L366K), 50 μM IPTG (red) for Lpp(C21G), and 50 μM IPTG + 0.2% Arabinose (purple) for both Lpp(C21G) and DnaA(L366K) expression in M9+CAA+Glu media. **(B)** Serial dilutions of cutC-null cells, capable of expressing Lpp(C21G) and DnaA(L366K), on agar plates. **(C,D)** Immunoblotting for the detection of DnaA(L366K) and Lpp(C21G) and quantitative analysis of Lpp(C21G) levels. **(E)** Viability of cutC-null cells expressing DnaA(L366K) and Lpp. No inducer control (black), in the presence of 0.2% Arabinose (green) for DnaA(L366K), 50 μM IPTG (red) for Lpp, and 50 μM IPTG + 0.2% Arabinose (purple) for both Lpp and DnaA(L366K) expression in M9+CAA+Glu media. **(F)** Serial dilutions of cutC-null cells, capable of expressing plasmid-derived Lpp and DnaA(L366K), on agar plates. **(G,H)** Immunoblotting for the detection of DnaA(L366K) and Lpp and quantitative analysis of Lpp levels. Lpp(C21G) and Lpp are expressed via Lpp (lpp^*P*^) and Lac (lacUV5^*OP*^) promoters. All loading controls are ponceau-S staining for total protein normalization presented in grayscale. Viability is expressed in cfu/ml and presented on a linear scale. Data are means ± SEM of at least three independent experiments. ^∗^*p* < 0.05, ns *p* > 0.05 in one-way ANOVA with Dunnett’s multiple comparison test.

In *cutC-null* cells, we observed an approximate 250-fold reduction (*p* < 0.02) in cell viability (Figures 3A,B) by the limited (50 μM IPTG) expression of Lpp(C21G) as compared to that for cells not induced for Lpp(C21G) expression. In line with this observation, we co-expressed in *cutC-null* cells Lpp(C21G) along with the DnaA(L366K). DnaA(L366K) overproduction resulted in an approximate 16-fold increase in cell viability, even when Lpp(C21G) expression was induced (Figure 3A). Although, in comparison with *cutC*^+^ cells (Figures 1C,D), *cutC-null* cells co-expressing Lpp(C21G) and DnaA(L366K) showed limited capacity to restore cell viability (Figures 3A,B).

The immunoblotting analysis further showed no significant decrease in levels of Lpp(C21G) in *cutC-null* cells in the absence or presence of DnaA(L366K) (lanes 3–4, Figures 3C,D), as opposed to cells with *cutC*^+^ cells (lanes 4–5, Figures 2C,D). Of note, we also observed a limited expression of Lpp(C21G) (lane 1, Figure 3C) from the *lpp* promoter in cells with DnaA(L366K). Interestingly, with the wild-type Lpp control, we witnessed no change in viability (Figures 3E,F). Moreover, there were no significant differences in Lpp expression in the presence or absence of DnaA(L366K) overexpression (lanes 4–5, Figures 3G,H), indicating that DnaA(L366K) selectively decreases the expression of Lpp(C21G), but not Lpp, in a σ^*E*^-dependent MicL-S-mediated manner.

### Δ*fis* Cells Show the Ability to Grow Even When the Lpp(C21G) Expression Is Induced

The capacity of DnaA(L366K) to still rescue growth to some extent in the absence of MicL-S sRNA led us to then question whether the lack of acidic phospholipid-mediated accumulation of pro-lipoprotein and the ectopic expression of Lpp(C21G) poisons the DnaA-dependent, *oriC*-mediated DNA replication initiation process. Considering that DNA replication occurs in the vicinity of inner cellular membranes, where the outer membrane lipoprotein accumulates due to faulty maturation, we wondered whether the accumulated, unprocessed pro-Lpp intermediate creates stress on the chromatin structure, causing growth arrest linked to DNA replication. To characterize the adverse effect of Lpp(C21G) on the initiation, we employed a loss-of-function study targeting NAPs that participate in DnaA-dependent initiation of DNA replication at *oriC* (Riber et al., 2016). *E. coli* strains lacking gene-encoding regions for HU-α, HU-β, Ihf-A, Ihf-B, Fis, and SeqA (Supplementary Table 1) were used to see which of those proteins might be adversely affected by the accumulation of the Lpp intermediate by testing which NAP deletions allowed growth upon the overproduction of Lpp(C21G). Cells lacking HU-α, HU-β, Ihf-A, Ihf-B, and SeqA (Supplementary Figures 3A–J) were still adversely affected by the overproduction of Lpp(C21G), thus suggesting that the Lpp(C21G) does not directly affect these NAPs. In contrast, the cells lacking Fis were able to retain approximately 84 and 64% viability even when Lpp(C21G) expression was induced with IPTG at 50 and 1,000 μM, respectively. It was noted, however, that colonies were large, opaque, and irregularly lobate (Supplementary Figures 3K,L).

**FIGURE 4:**
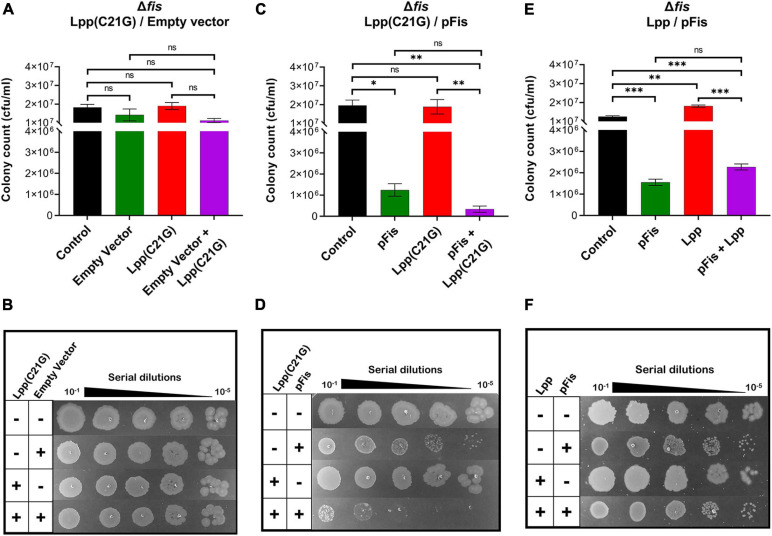
Cells lacking fis can grow even when the inducer for Lpp(C21G) is present. **(A)** Viability of *fis-null* cells transformed with empty vector and plasmids expressing Lpp(C21G). No inducer control (black), in the presence of 0.2% Arabinose (green) for empty vector, 50 μM IPTG (red) for Lpp(C21G), and 50 μM IPTG + 0.2% Arabinose (purple) for both Lpp(C21G) and empty vector expression in M9+CAA+Glu media. **(B)** Serial dilutions of *fis-null* cells, capable of expressing plasmid-derived Lpp(C21G) and empty vector, on agar plates. **(C)** Viability of *fis-null* cells expressing Fis (pFis) and Lpp(C21G). No inducer control (black); in the presence of 0.2% Arabinose (green) for Fis, 50 μM IPTG (red) for Lpp(C21G), and 50 μM IPTG + 0.2% Arabinose (purple) for both Lpp(C21G) and Fis expression in M9+CAA+Glu media. **(D)** Serial dilutions of *fis-null* cells, capable of expressing plasmid-derived Lpp(C21G) and Fis on agar plates. **(E)** Viability of *fis-null* cells expressing Fis (pFis) and Lpp. No inducer control (black), in the presence of 0.2% Arabinose (green) for Fis, 50 μM IPTG (red) for Lpp, and 50 μM IPTG + 0.2% Arabinose (purple) for both Lpp and Fis expression in M9+CAA+Glu media. **(F)** Serial dilutions of *fis-null* cells, capable of expressing plasmid-derived Lpp and Fis, on agar plates. Viability is expressed in cfu/ml and presented on a linear scale. Data are means ± SEM of at least three independent experiments. ^∗^*p* < 0.05, ^∗∗^*p* < 0.01, ^∗∗∗^*p* < 0.001, ns *p* > 0.05 in one-way ANOVA with Dunnett’s multiple comparison test.

A two-plasmid system introduced Fis into *fis*-null cells expressing Lpp(C21G). As was expected, no adverse effects of Lpp(C21G) on cell viability (Figures 4A–D) were observed in the *fis-null* cells. However, when Fis was exogenously expressed in cells also expressing Lpp(C21G), a 57.4-fold (*p* < 0.006) loss of cell viability (Figures 4C,D) was observed. We also noticed that the ectopic expression of Fis alone led to a 15.7-fold (*p* < 0.01) reduction in cell viability (Figure 4C). However, the combined expression of Lpp(C21G) and Fis [pFis+Lpp(C21G), Figure 4C] had a more severe effect on viability than the expression of Fis alone (pFis, Figure 4C: approximately fourfold reduction). As a control, we tested the effect of Fis in cells expressing wild-type Lpp. Cell viability (Figures 4E,F) remained unaffected following the overexpression of Lpp alone. Like cells expressing Lpp(C21G), the overexpression of Fis alone affected viability in *fis-null* cells carrying a plasmid for Lpp. The co-expression of Lpp and Fis (pFis+Lpp, Figure 4E) resulted in increased viability when compared with Fis expression alone (pFis, Figure 4E), as opposed to the co-expression of Lpp(C21G) and Fis [pFis+Lpp(C21G), Figure 4C].

### Cells Lacking Fis Have Reduced Levels of Lpp(C21G) but Not Lpp

**FIGURE 5:**
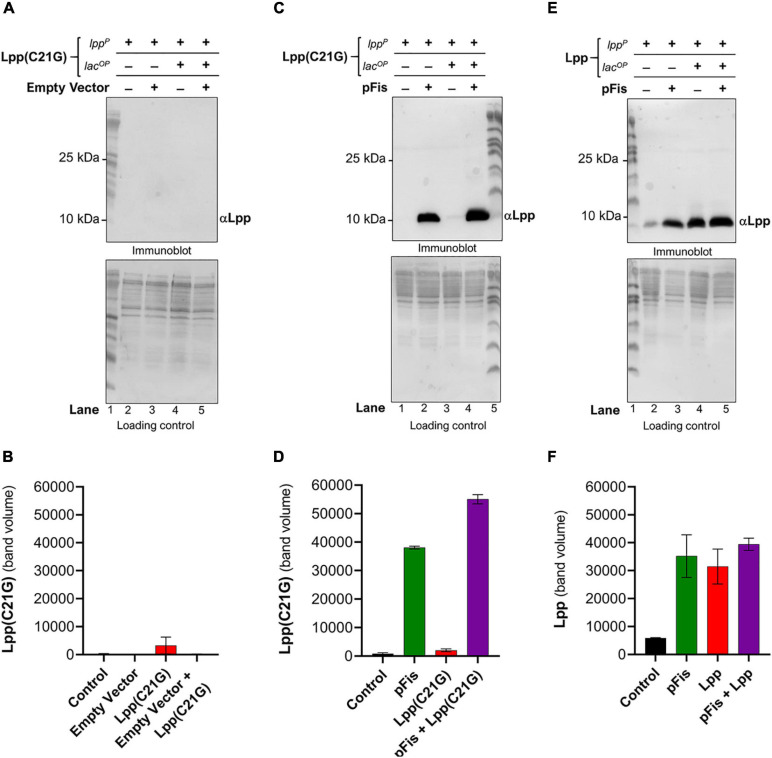
Cells lacking fis reduces levels of Lpp(C21G) even when the inducer is present. **(A,B)** Immunoblotting of *fis-null* cells carrying an empty vector and a plasmid expressing Lpp(C21G) to detect Lpp(C21G) and quantitative analysis of Lpp(C21G) levels. **(C,D)** Immunoblotting of *fis-null* cells carrying plasmid expressing Fis (pFis) and Lpp(C21G) to detect Lpp(C21G) and quantitative analysis of Lpp(C21G) levels. **(E,F)** Immunoblotting of *fis-null* cells carrying plasmid expressing Fis (pFis) and Lpp to detect Lpp and quantitative analysis of Lpp levels. Lpp(C21G) and Lpp are expressed *via* Lpp (*lpp*^*P*^) and Lac (*lacUV5^*OP*^*) promoters. All loading controls are ponceau-S staining for total protein normalization presented in grayscale. Data are means ± SEM of at least three independent experiments.

Seeing that the DnaA(L366K) reduces levels of Lpp(C21G) but not the wild-type Lpp, as mentioned above, we wanted to test whether *fis-null* cells restore growth to Lpp(C21G)-expressing cells, in a manner similar to the ectopic expression of DnaA(L366K), by reducing Lpp(C21G) expression but not Lpp. Surprisingly, in the cells carrying vector control, we did not detect any Lpp(C21G) when expressed from either the *lpp* promoter (lanes 2–3, Figures 5A,B) and only limited expression of Lpp(C21G) was seen from the combined *lpp* and *lacUV5* promoters (lanes 4–5, Figures 5A,B). The expression of Lpp(C21G) was evident only in the presence of Fis (lanes 2 and 4, Figures 5C,D). In contrast, for cells that carry plasmids for Fis and wild-type Lpp, we detected Lpp from both *lpp* (lanes 2–3, Figures 5E,F) and combined *lpp* and *lacUV5* promoters (lanes 4–5, Figures 5E,F), as opposed to Lpp(C21G) (lane 3, Figures 5C,D). We also noted that in cells lacking *fis*, ectopic expression of Lpp through the *lpp* promoter (lanes 2–3, Figure 5E) was similar to that caused by DnaA(L366K) (lanes 2–3, Figure 2E).

### *fis*-*null* Cells Lacking the Promoter Region for MicL-S Small RNA Only Partially Express Lpp(C21G) but Still Are Able to Grow

**FIGURE 6:**
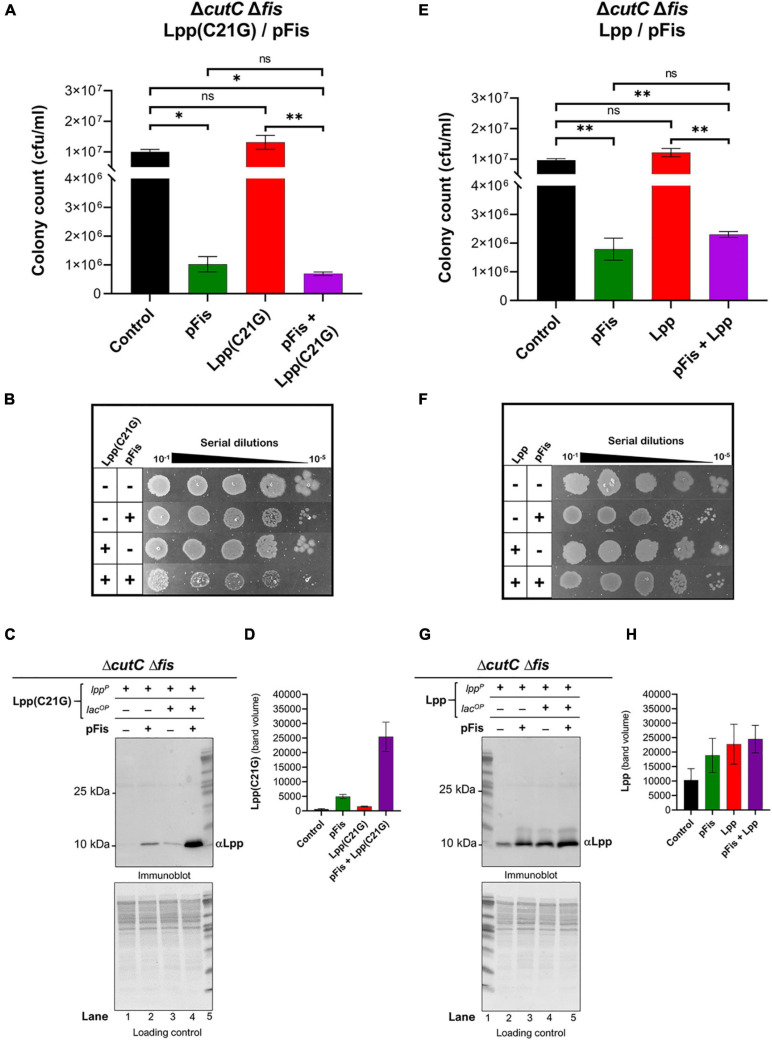
*fis-null* cells lacking promoter for MicL-S small RNA can grow by expressing only low levels Lpp(C21G). **(A)** Viability of *cutC-null fis-null* cells expressing Fis (pFis) and Lpp(C21G). No inducer control (black); in the presence of 0.2% Arabinose (green) for Fis, 50 μM IPTG (red) for Lpp(C21G), and 50 μM IPTG + 0.2% Arabinose (purple) for both Lpp(C21G) and Fis expression in M9+CAA+Glu media. **(B)** Serial dilutions of *cutC-null fis-null* cells, capable of expressing plasmid-derived Lpp(C21G) and Fis, on agar plates. **(C,D)** Immunoblotting for the detection of Lpp(C21G) and quantitative analysis of Lpp(C21G) levels. **(E)** Viability of *cutC-null fis-null* cells expressing Fis (pFis) and Lpp. No inducer control (black), in the presence of 0.2% Arabinose (green) for Fis, 50 μM IPTG (red) for Lpp, and 50 μM IPTG + 0.2% Arabinose (purple) for both Lpp and Fis expression in M9+CAA+Glu media. **(F)** Serial dilutions of *cutC-null fis-null* cells, capable of expressing plasmid-derived Lpp and Fis, on agar plates. **(G,H)** Immunoblotting for the detection of Lpp and quantitative analysis of Lpp levels. Lpp(C21G) and Lpp are expressed *via* Lpp (*lpp*^*P*^) and Lac (*lacUV5^*OP*^*) promoters. All loading controls are ponceau-S staining for total protein normalization presented in grayscale. Viability is expressed in cfu/ml and presented on a linear scale. Data are means ± SEM of at least three independent experiments. ^∗^*p* < 0.05, ^∗∗^*p* < 0.01, ns *p* > 0.05 in one-way ANOVA with Dunnett’s multiple comparison test.

We examined the role of MicL-S small RNA in the reduction of Lpp(C21G) levels in *fis*-*null* cells. In cells lacking Fis and the promoter region for MicL-S small RNA (Δ*cutCΔfis*, Supplementary Table 1), induction of Lpp(C21G) (Figures 6A,B) and Lpp (Figures 6E,F) alone did not adversely affect cell viability, in a manner similar to *cutC^+^ fis-null* cells (Figures 4C–F). Interestingly, immunoblotting analysis detected an approximate 1.8-fold higher expression of Lpp(C21G) (lane 3, Figures 6C,D) when expressed alone, as opposed to that in *cutC^+^ fis-null* cells (lane 3, Figures 5C,D). However, the levels of Lpp(C21G) in *cutC-null, fis-null* cells (lane 3, Figures 6C,D) were still 16-fold less in comparison with the co-expression of Lpp(C21G) and Fis (lane 4, Figures 6C,D).

The ectopic expression of Fis alone still caused growth inhibition (Figures 6A,E). At the same time, co-expression of Fis and Lpp(C21G) caused a 14.5-fold reduction (*p* = 0.02, Figure 6A) in the cell viability, as opposed to a 4.2-fold decrease (*p* = 0.009, Figure 6E) when Fis and wild-type Lpp were co-expressed. Intriguingly, expression of Lpp was unaffected by the lack of *fis* (lanes 3–5, Figures 5E,F) or *fis* and *cutC* (lanes 3–5, Figures 6G,H), as was seen for cells expressing DnaA(L366K) (lanes 3–5, Figures 2E,F), showing a mutation-specific decrease in the gene expression of the Lpp(C21G) but not Lpp.

### *fis-null* Cells Unable to Synthesize PG and CL Are Viable

**FIGURE 7:**
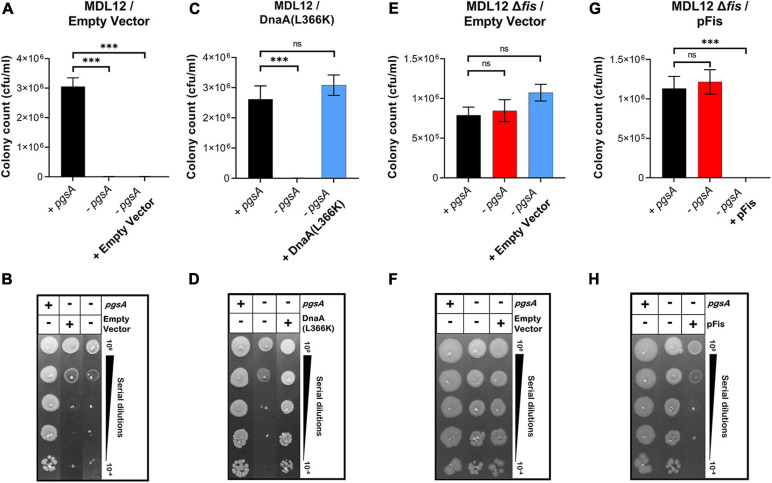
Lack of fis in cells deficient in phosphatidylglycerol (PG) synthesis permits growth. **(A,B)** Viability of MDL12 cells carrying empty vector with 1 mM IPTG for the expression of *pgsA* (black), no inducer control (red), and 0.2% Arabinose control (blue) in M9+CAA+Glu media. **(C,D)** Viability of MDL12 cells carrying plasmid for DnaA(L366K) with 1 mM IPTG for the expression of *pgsA* (black), no inducer control (red), 0.2% Arabinose for DnaA(L366K) (blue) in M9+CAA+Glu media. **(E,F)** Viability of MDL12 *fis-null* cells carrying empty vector with 1 mM IPTG for the expression of *pgsA* (black), no inducer control (red), and 0.2% Arabinose control (blue) in M9+CAA+Glu media. **(G,H)** Viability of MDL12 *fis-null* cells carrying plasmid for Fis (pFis) with 1 mM IPTG for the expression of *pgsA* (black), no inducer control (red), and 0.2% Arabinose for Fis (blue) in M9+CAA+Glu media. Viability is expressed in cfu/ml and presented on a linear scale. Data are means ± SEM of at least three independent experiments. ^∗∗∗^*p* < 0.001, ns *p* > 0.05 in one-way ANOVA with Dunnett’s multiple comparison test.

Previously, it was seen that the ectopic expression of DnaA(L366K) allows cell growth even when acidic phospholipids are absent. We now questioned whether deletion of *fis* permitted growth in *lpp*^+^ cells lacking normal levels of acidic phospholipids. *E. coli* MDL12 cells, which have chromosomal *pgsA* under control of the *lac* promoter, undergo growth arrest in the absence of inducer (IPTG) due to diminished levels of anionic phospholipids (Fingland et al., 2012; Figures 7A,B). MDL12 *fis-null* cells (Supplementary Table 1) were able to grow even when the inducer IPTG was absent (Figures 7E,F). To confirm that this outcome was due to the loss of Fis, we exogenously expressed Fis in MDL12 *fis-null* cells. We noted that the ectopic expression of Fis indeed resulted in severely diminished viability (Figures 7G,H) when acidic phospholipid biosynthesis was not induced.

**FIGURE 8:**
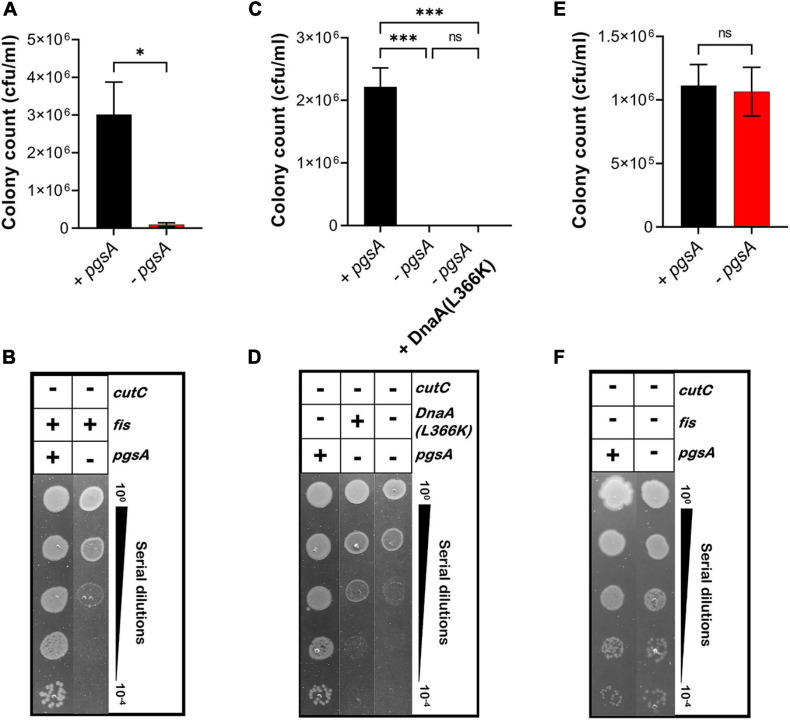
Growth of *fis-null* cells lacking promoter for MicL-S small RNA does not depend on cellular levels of acidic phospholipids. **(A,B)** Viability of MDL12 *cutC-null* cells treated with 1 mM IPTG for the expression of *pgsA* (black), no inducer control (red) in M9+CAA+Glu medium. **(C,D)** Viability of MDL12 *cutC-null fis-null* cells treated with 1 mM IPTG for the expression of *pgsA* (black), no inducer control (red) in M9+CAA+Glu medium. **(E,F)** Viability of MDL12 *cutC-null* cells carrying plasmid for DnaA(L366K) with 1 mM IPTG for the expression of *pgsA* (black), no inducer control (red), and 0.2% Arabinose control (blue) in M9+CAA+Glu medium. Viability is expressed in cfu/ml and presented on a linear scale. Data are means ± SEM of at least three independent experiments. ^∗^*p* < 0.05, ^∗∗∗^*p* < 0.001, ns *p* > 0.05 in Student’s *t*-test for analysis in plots A and C, and one-way ANOVA with Dunnett’s multiple comparison test.

Furthermore, we examined whether the growth-arrested phenotype of *E. coli* MDL12 cells lacking *cutC* gene could be suppressed by either overexpression of DnaA(L366K) or deletion of chromosomal *fis*. Our result suggested that the growth of cells lacking the chromosomal *cutC* allele is dependent on cellular levels of acidic phospholipids (Figures 8A,B). Whereas cells carrying the intact *pgsA* allowed growth when *cutC* is not present, the absence of *pgsA* allele adversely affected growth of cells lacking *cutC* (Figures 8A,B). Moreover, overexpression of DnaA(L366K) cannot suppress the growth-arrested phenotype when *cutC*-null cells are unable to synthesize acidic phospholipids (Figures 8C,D). In contrast, removal of the chromosomal *fis* allele allowed growth in a *pgsA* independent manner (Figures 8E,F). These results agree with our findings that either the cells overexpressing mutant Lpp protein or the lack of cellular levels of acidic phospholipids resulted in the accumulation of cytotoxic form of Lpp(C21G), causing a growth-arrested phenotype.

### *fis-null* Cells Unable to Synthesize PG Have an Altered Lipidomic Profile

**FIGURE 9:**
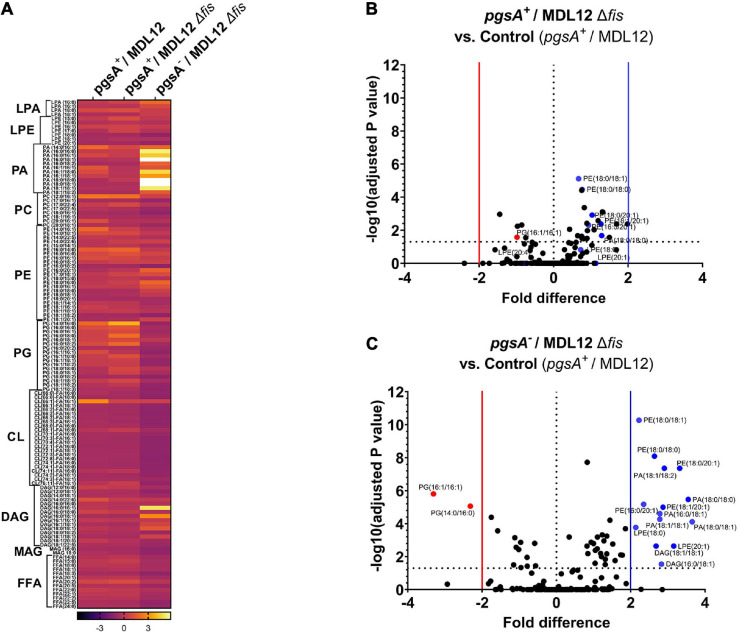
Lipidomic analysis of MDL12 cells in the presence or absence of Fis. **(A)** Lipidomic analysis represented in heatmap comparing MDL12 cells with 1 mM IPTG for the expression of *pgsA* (*pgsA*^+^/MDL12), MDL12 *fis-null* cells with 1 mM IPTG for the expression of *pgsA* (*pgsA*^+^/MDL12Δ*fis*), and MDL12 *fis-null* cells without expression of *pgsA* (*pgsA^–^ /*MDL12Δ*fis*). Each row indicates the ratio values of individual lipid species for at least three biological replicates. ANOVA was performed to compare statistical differences between each group. Volcano plots comparing distribution of 191 lipids species among. **(B)** MDL12 *fis-null* cells expressing *pgsA* (*pgsA*^+^/MDL12Δ*fis*) and control [MDL12 cells expressing of *pgsA* (*pgsA*^+^/MDL12)]. **(C)** MDL12 *fis-null* cells *without* expression of *pgsA* (*pgsA^–^ /*MDL12Δ*fis*) and control [MDL12 cells expressing of *pgsA* (*pgsA*^+^/MDL12)]. Horizontal dotted line indicates statistical significance with *p* = 0.05 for at least three biological replicates. A vertical red line separates red dots indicating data points with statistically significant fold decrease. A vertical blue line separates blue dots, indicating data points with statistically significant fold increase.

We assessed the lipid profile of MDL12 cells with and without Fis to confirm that the absence of Fis did not somehow bestow a normal lipid composition. A targeted LC-MS approach to estimate total lipid content detected no evident change in phospholipid content in *pgsA-*expressing cells with or without endogenous levels of Fis (Figure 9A). However, in cells lacking *fis* and *pgsA*, we observed that out of 191 lipid metabolites, 60 phospholipid species were differentially produced as compared to *fis*^+^ and *pgsA*^+^ cells with levels of PG significantly reduced in the absence of active *pgsA* and *fis* (Figures 9B,C). The depletion in the levels of PG was accompanied by significantly higher levels of phosphatidic acid (PA), phosphatidylethanolamine (PE), diacylglycerol (DAG), and lysophosphatidylethanolamine (LPE) in comparison with the *fis*^+^ and *pgsA*^+^ cells.

## Discussion

**FIGURE 10:**
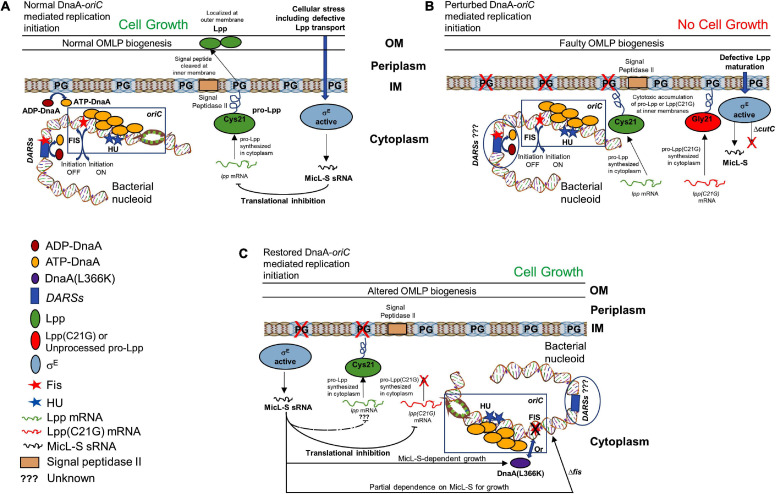
Schematic representation of how membrane stress caused by unprocessed outer membrane lipoprotein intermediate pro-Lpp affects DnaA and Fis-dependent growth. **(A)** Cells carrying normal cellular levels of inner membrane acidic phospholipids, PG, and CL support biogenesis of outer membrane lipoprotein (Lpp) and initiate DNA replication at the chromosomal origins. ATP-DnaA binds at specific *oriC* sites, and in the presence of HU protein, unwinds the duplex DNA, leading to the formation of a functional replisome. Acidic membranes and the specific chromosomal site DARSs play a role in the rejuvenation of replication-defective ADP-DnaA to replication-proficient ATP-DnaA. Binding of Fis protein at *oriC* and DARSs helps maintain timely inhibition of replication initiation. Stress to the cellular envelope stimulates the activation of an alternative sigma factor (such as sigma factor E, σ^*E*^), which regulates the production of Lpp protein through MicL-S sRNA in response to the defective Lpp localization. **(B)** Cells unable to synthesize PG or that accumulate cytotoxic mutant Lpp(C21G) on the inner membrane are negatively affected for growth. **(C)** Cells expressing Lpp(C21G) or lacking PG can grow in a σ^*E*^-dependent MicL-S sRNA manner, when overexpressing DnaA(L366K). In addition, restoration of growth can occur in cells lacking *fis*, independently of MicL-S sRNA.

Cellular membranes have long been hypothesized to be involved in chromosomal replication, including accumulating evidence indicating that membranes have an influence on DnaA protein function. *E. coli pgsA*-null cells unable to synthesize PG and CL contain decreased chromosomal DNA content due to reduced frequency of initiation from chromosomal origin and thus undergo growth-arrested phenotype (Fingland et al., 2012). In addition, cells deficient in PG and CL are unable to process outer membrane lipoprotein intermediates, with the intermediates accumulating at the inner membrane. Alternatively, defective lipoprotein sorting in bacterial cells containing an intact *pgsA* allele can be achieved by overproducing mutant Lpp protein in which the Cys-21 residue is substituted with Gly (Figures 10A,B). Although two not obviously related processes, initiation of replication and the biogenesis of lipoproteins are associated with the cellular composition of bacterial membranes, and any linkage among them is unknown, yet. We hypothesized the possibility that unprocessed pro-Lpp intermediate might poison the DnaA–*oriC*-mediated process of replication of initiation.

A specific point mutant of chromosomal replication initiator protein, DnaA(L366K), when working in concert with wild-type DnaA, can initiate DNA synthesis both *in vivo* (Zheng et al., 2001) and *in vitro* (Saxena et al., 2011) and suppress the arrested growth of *pgsA* null cells. However, any mechanistic insights into how that occurs remain unknown. Secondly, the inability of cells to synthesize acidic membranes results in accumulation of unprocessed pro-lpp protein. That leads to the question of whether accumulating, unprocessed pro-Lpp poisons DnaA-dependent replication. Therefore, a valid question to ask is whether DnaA(L366K) can allow growth in cells carrying mutant Lpp(C21G). We found that the bacterial cells expressing both DnaA(L366K) and Lpp(C21G) (Figures 1C,D) can survive, but only with a marked decrease in the expression of the latter (lane 5, Figures 2C,D). There is evidence that the cellular abundance of Lpp is regulated by RNA polymerase sigma factor σ^*E*^-dependent, small RNA, MicL-S (Guo et al., 2014). This led us to further test if cells overexpressing mutant Lpp(C21G) but lacking the σ^*E*^-dependent MicL-S-mediated regulatory loops are capable of growth. If so, it would seem that DnaA(L366K) contains the potential to override cellular toxicity independent of MicL-S. Our results indicate that overexpression of DnaA(L366K) can bestow partial growth when Lpp(C21G) is present in growth-arresting amounts even when the σ^*E*^-dependent envelope-protective mechanism is inactive (Figure 3A). Earlier work indicates that DnaA overproduction increases polA (encodes DNA polymerase I) expression in stationary-phase cultures (Quinones et al., 1997). The stimulation effect was independent of rpoS, which encodes the sigma factor for stationary-phase-inducible genes, which also include σ^*E*^ involved in the transcription of MicL.

From the subsequent loss-of-function study to determine whether Lpp(C21G) adversely affects the orisome, we found that cells lacking Fis were able to grow even when the inducer for Lpp(C21G) expression is present. However, the combined expression of Fis and Lpp(C21G) (Figures 4C,D) was highly inhibitory for growth in contrast to co-expression of Fis and Lpp (Figures 4E,F). These observations confirmed that Fis, at least when overproduced, is adversely affected by the inner membrane perturbed by accumulating Lpp intermediates. Strikingly, the near-complete depletion of Lpp(C21G) (lane 4, Figure 5A, and lane 3, Figure 5C) but not Lpp (lane 4, Figure 5E) in *fis-null* cells, similar to the ectopic expression of DnaA(L366K) (lane 4, Figure 2C, and lane 4, Figure 2E), underscores a concurrence in the restoration of growth with a decrease in expression of Lpp(C21G). DnaA(L366K), however, shows a strong dependence on σ^*E*^-mediated MicL-S-dependent envelope protective loop to decrease Lpp(C21G) expression (lane 4, Figure 3C), while *fis-null* cells exhibit only a partial dependence on MicL-S-mediated translational inhibition of Lpp(C21G) (lane 3, Figure 6C) and thus indicates the pleiotropic role of Fis.

Cells lacking Fis grow even when normal levels of PG are absent (Figures 7E–H), confirming that the perturbation of inner membranes affects growth in a Fis-dependent manner. Our targeted LC-MS lipidomic approach to determine changes in total phospholipid content in the presence or absence of Fis indicates a significant fold increase in PA and other phospholipid species such as PE, DAG, and LPE when both *pgsA* and *fis* are absent (Figures 9A–C). This observation agrees with a previous study involving the ectopic expression of DnaA(L366K) to rescue cell growth in PG-deficient cells, accumulating higher levels of PA (Zheng et al., 2001). However, the elevated levels of PA do not fully substitute for the normal combined levels of PG and CL (Heacock and Dowhan, 1987; Figures 7A,B). Intervention, such as (1) high levels of DnaA(L366K) (Zheng et al., 2001; Figures 7C,D) or (2) lack of Fis (Figures 7E–H), may serve to reorganize the orisome. In conclusion, we now suggest that the poisoning of orisome by unprocessed immature pro-lipoprotein present at the inner membranes causes growth inhibition when cells are lacking normal levels of acidic phospholipids (Figure 10). This report suggests an intricate network between the physiological state and the composition of bacterial membranes and the orisome for the proper initiation of the chromosomal DNA replication (Figure 10C).

The role of auxiliary replication initiation factor Fis is well-established as a global transcription factor undertaking several functions (Cho et al., 2008). In addition to acting as a global transcription factor, Fis, like other nucleoid-associated proteins, remains associated with bacterial nucleoid and serves as a DNA bending–binding protein that helps to maintain the proper confirmation of *oriC* DNA (Gille et al., 1991). The binding of Fis protein to *oriC* DNA prevents untimely initiation of DNA replication in the cells, therefore serving as a negative regulator of DnaA-mediated replication at *oriC* (Wold et al., 1996). At the time of initiation, Fis needs to be removed and replaced with DnaA protein to form replication-proficient DnaA–*oriC* complexes (Margulies and Kaguni, 1998). Certain synthetic *oriC* sequences carrying mutations in DnaA recognition sites as well as Fis or IHF binding sites cause asynchronous initiations (Weigel et al., 2001). In addition to binding *oriC*, Fis binding to *DARSs* may regulate timely initiation of replication (Kasho et al., 2014). It remains to be determined whether the lack of *fis* might alter any ongoing primary or secondary mechanisms required to maintain cellular levels of replication-proficient ATP-DnaA, and help bacterial cells to escape membrane-mediated cytotoxicity. Considering the important roles that Fis plays in both nucleoid structure and gene regulation, further studies are also needed to see how these roles are impacted in cells either stressed from insufficient acidic phospholipid levels or accumulating Lpp intermediates. These studies include how the cellular level of Fis protein controls the expression of the σ^*E*^ sigma factor, the processing of the small RNA such as MicL-S, and the levels and function of other important proteins such as different cellular proteases, which are known to upregulate when cells are stressed.

## Data Availability Statement

The original contributions presented in the study are included in the article/Supplementary Material, further inquiries can be directed to the corresponding author/s.

## Author Contributions

RS and DP conceived the idea along with EC. DP, RS, and DX performed experiments. MS provided the assistance. DP, RS, and EC analyzed the results and wrote the manuscript. DP, SB, and AC performed and analyzed lipidomic experiments. All authors have reviewed the results and approved the final version of the manuscript.

## Conflict of Interest

The authors declare that the research was conducted in the absence of any commercial or financial relationships that could be construed as a potential conflict of interest.

## Footnotes

1 http://www.generunner.net/

2 https://cgsc.biology.yale.edu
