# Supplementary material for: Membrane Stress Caused by Unprocessed Outer Membrane Lipoprotein Intermediate Pro-Lpp Affects DnaA and Fis-Dependent Growth
Source: Front Microbiol. 2021 Jun 7;12:677812. doi: 10.3389/fmicb.2021.677812 (PMC8216713; doi:10.3389/fmicb.2021.677812)
Supplement: Supplementary file 1 [file Data_Sheet_1.pdf]

Membrane-stress caused by unprocessed outer membrane lipoprotein intermediate, pro-Lpp affects DnaA and Fis-dependent growth

Supplementary Material

Supplementary Figure 1: Ectopic expression of Lpp(C21G) inhibits growth.

(A) Growth, and **(B)** Viability of *lpp-null* cells with an increasing concentration of IPTG with 0 μM (black), 10 μM (green), 50 μM (red), 100 μM (purple), and 1000 μM (orange) for the expression of Lpp in M9+CAA+Glu media. **(C)** Growth, and **(D)** Viability of *lpp-null* cells in the presence of an increasing concentration of IPTG with 0 μM (black), 10μM (green), 50 μM (red), 100 μM (purple), and 1000 μM (orange) for the expression of Lpp(C21G) in M9+CAA+Glu media. Viability is expressed in cfu/ml and presented on a linear scale. Bar denotes a standard error. Data are means ±SEM of at least three independent experiments. ***$p<0.001$, ns $p>0.05$ in one-way ANOVA with Dunnett's multiple comparison test. Serial dilutions of *lpp-null* cells spotted on varying concentrations of IPTG to induce **(E)** Lpp and **(F)** Lpp(C21G) on M9+CAA+Glu agar plates. **(G)** Immunoblotting analysis to compare plasmid-derived Lpp in *lpp-null* cells with endogenous Lpp (ten-fold dilution) from BW25113. **(H)** Immunoblotting for the detection of Lpp(C21G). Loading controls for immunoblotting are ponceau-S staining for total protein normalization presented in greyscale.

Supplementary Figure 2: Overexpression of DnaA fails to alleviate growth inhibition due to Lpp(C21G).

(A) Viability of *lpp-null* cells expressing DnaA and Lpp(C21G). No inducer control (black), in the presence of 0.2% Arabinose (green) for DnaA, 50 μM IPTG (red) for Lpp(C21G), and 50 μM

IPTG + 0.2% Arabinose (purple) for both Lpp(C21G) and DnaA expression in M9+CAA+Glu media. **(B)** Serial dilutions of *lpp-null* cells capable of expressing plasmid-derived Lpp(C21G) and DnaA on agar plates. **(C)** Immunoblotting for the detection of DnaA and Lpp(C21G). Loading control for immunoblotting is ponceau-S staining for total protein normalization presented in greyscale. Viability is expressed in cfu/ml and presented on a linear scale. Data are means ±SEM of at least three independent experiments. ****$p<0.001$,* in one-way ANOVA with Dunnett's multiple comparison test.

Supplementary Figure 3: Lack of either HU, IHF, or SeqA unable to alleviate inhibited growth due to overexpression of Lpp(C21G)

(A) Viability, and **(B)** Serial dilutions of *hupA-null lpp-null* cells expressing Lpp(C21G). No inducer control (black), and in the presence of 50µM IPTG (red) for Lpp(C21G) expression in M9+CAA+Glu media. **(C)** Viability, and **(D)** Serial dilutions of *hupB-null lpp-null* cells expressing Lpp(C21G). No inducer control (black), and in the presence of 50µM IPTG (red) for Lpp(C21G) expression in M9+CAA+Glu media. **(E)** Viability, and **(F)** Serial dilutions of *ihfA-null* cells expressing Lpp(C21G). No inducer control (black), and in the presence of 50µM IPTG (red) for Lpp(C21G) expression in M9+CAA+Glu media. **(G)** Viability, and **(H)** Serial dilutions of *ihfB-null* cells expressing Lpp(C21G). No inducer control (black), and in the presence of 50µM IPTG (red) for Lpp(C21G) expression in M9+CAA+Glu media. **(I)** Viability, and **(J)** Serial dilutions of *seqA-null lpp-null* cells with plasmids expressing Lpp(C21G). No inducer control (black), and in the presence of 50µM IPTG (red) for Lpp(C21G) expression in M9+CAA+Glu media. **(K)** Viability, and **(L)** Serial dilutions of *fis-null lpp-null* cells with plasmids expressing Lpp(C21G). No inducer control (black); in the presence of 50 µM IPTG (red), 1000 µM IPTG (blue) for Lpp(C21G) expression in M9+CAA+Glu media. Viability is expressed in cfu/ml and presented on a linear scale. Data are means ±SEM of at least two independent experiments.

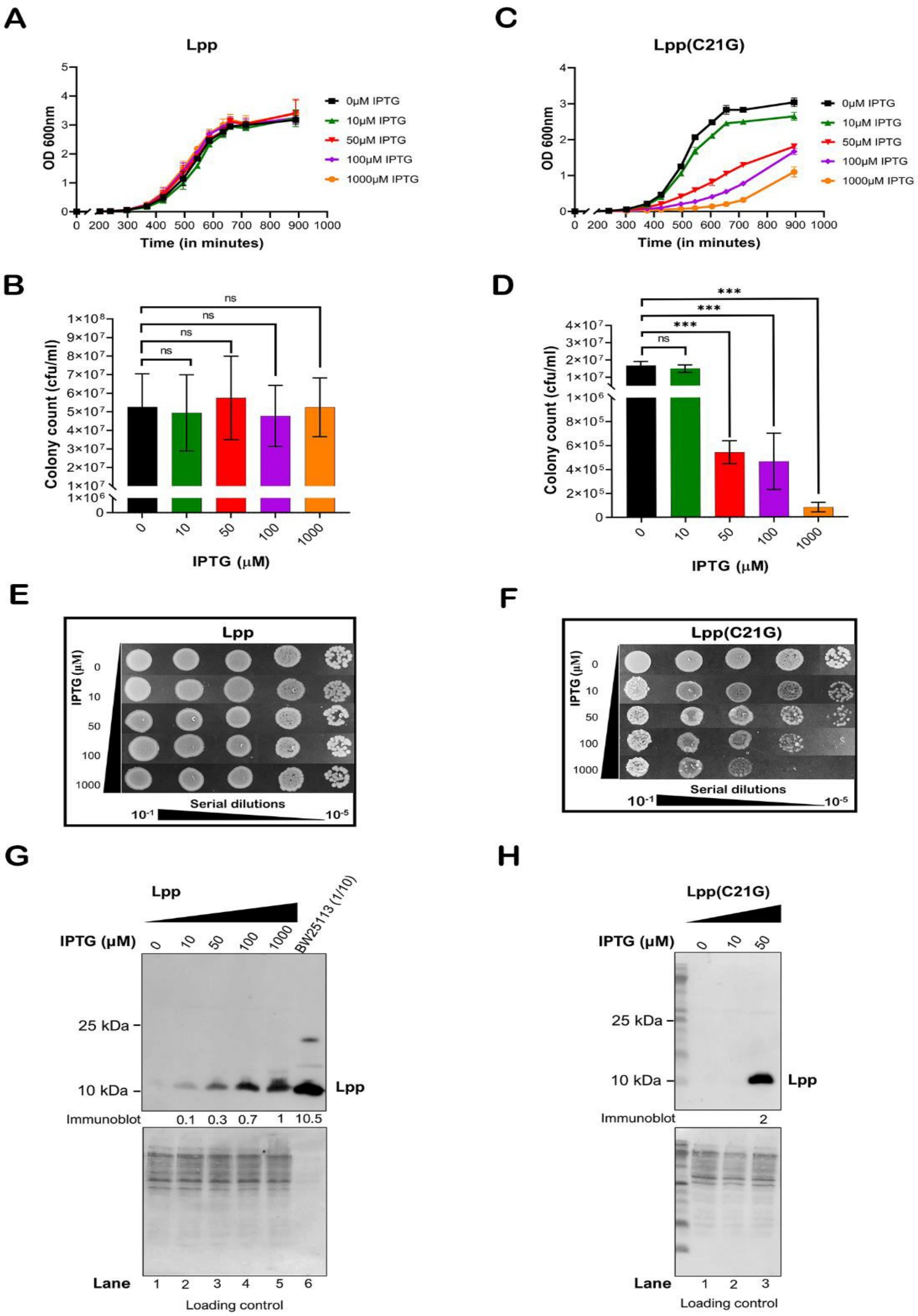

Supplementary Figure 1

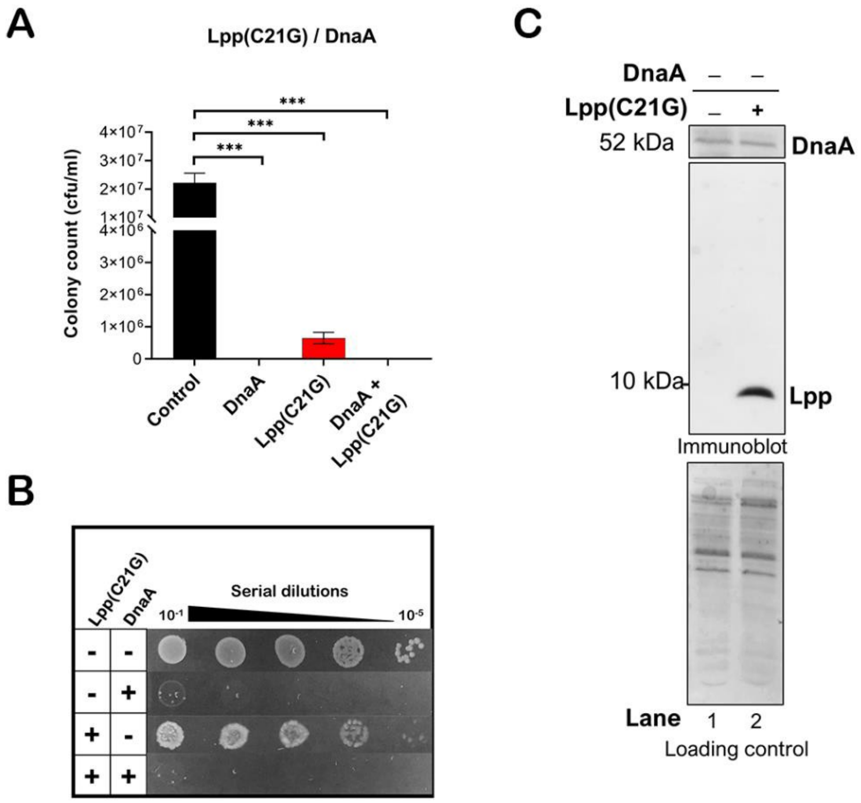

Supplementary Figure 2

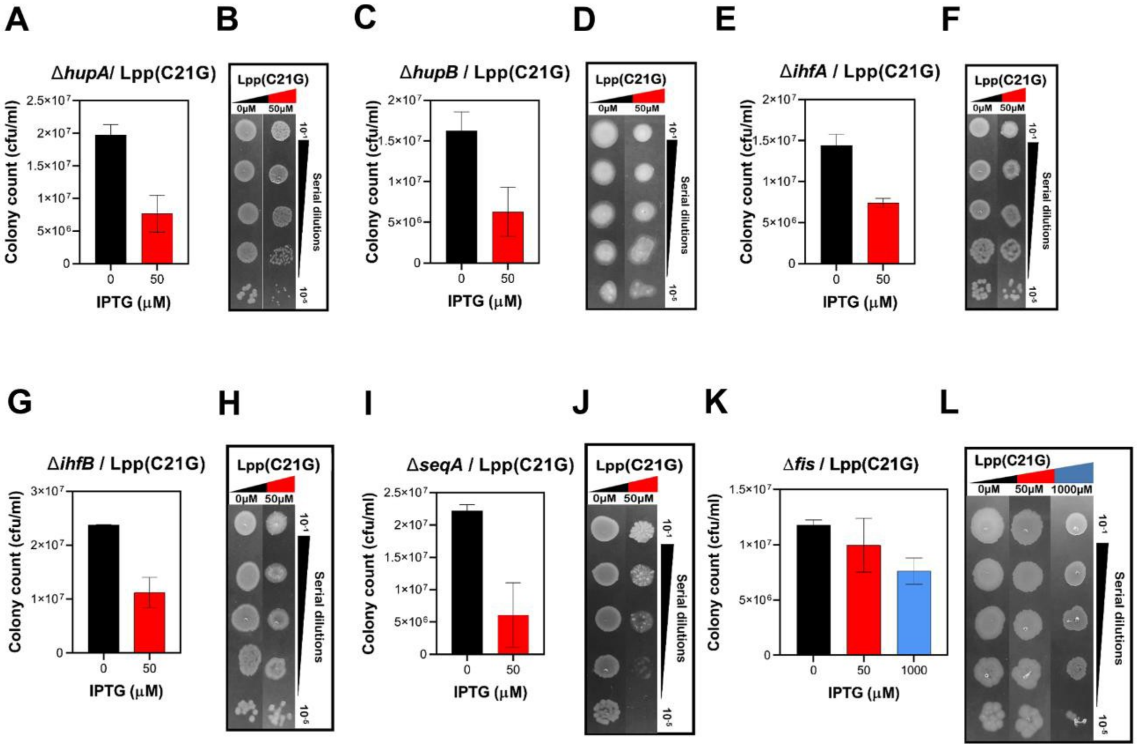

Supplementary Figure 3

Supplementary Table 1

Strain	Genotype	Source or Reference
BW25113	F-, *Δ[araD-araB]567*, *ΔlacZ4787*[::rrnB-3], λ^-, *rph-1*, *Δ[rhaD-rhaB]568*, *hsdR514*, $lacI^+$	Datsenko et al., 2000
JW1667-5	BW25113, *Δlpp-752::kan*,	Baba et al., 2006
DAP1001	JW1667-5, *Δfis::cam*	This study
JW3964-1	BW25113, *ΔhupA771::kan*	Baba et al., 2006
DAP1002	JW3964-1, *Δlpp::cam*	This study
JW0430-3	BW25113, *ΔhupB726::kan*	Baba et al., 2006
DAP1003	JW3964-1, *Δlpp::cam*	This study
JW1702-1	BW25113, *ΔihfA786::kan*	Baba et al., 2006
JW0895-3	BW25113, *ΔihfB735::kan*	Baba et al., 2006
JW0674-1	BW25113, *ΔseqA735::kan*	Baba et al., 2006
DAP1004	JW0674-1, *Δlpp::cam*	This study
JW3229-1	BW25113, *Δfis779::kan*	Baba et al., 2006
JW1683-1	BW25113 *ΔcutC728::kan*,	Baba et al., 2006
DAP1005	JW1683-1, *Δlpp::cam*	This study
DAP1006	JW1683-1, *Δfis::spec Δlpp::cam*	This study
MDL12	MG1655 *pgsA30::kan* , F*[lacOP-pgsA+]1 lacZ'*, *lacY*::Tn9	Xia et al., 1995
DAP1007	MDL12, *Δfis::spec*	This study
DAP1008	MDL12, *ΔcutC::tet*	This study
DAP1009	MDL12, *Δfis::spec*, *ΔcutC::tet*	This study
DH5α	F-, Δ[*argF-lac*]169, φ80d*lacZ58*[M15], Δ*phoA8*, *glnX44*[A_S], λ-, *deoR*481, *rfbC*1, *gyrA96*[NalR], *recA1*, *endA1*, *thiE1*, *hsdR17*	Lab stock
**Plasmids**		
pC2	*pBR322, lacI*, Amp^R, lpp^P-lac^{PO} -$lppCys^{21}$-*Gly*	Inouye et al.,1983
pC2-Lpp	*pBR322, lacI*, Amp^R, lpp^P-lac^{PO} -*lpp*	This study
pSC	*p15A, araC*, Tet^R, P_{BAD}	This study
pSC(*dnaA*)	*p15A, araC*, Tet^R, P_{BAD}- *dnaA*	This study
pSC(*dnaAL366K*)	*p15A, araC*, Tet^R, P_{BAD}-*dnaA(L366K)*	This study
pSC-Fis	*p15A, araC*, Tet^R, P_{BAD}-*fis*	This study
pKD-sg-ack	*pSC101*, ori^{TS}, *araC*, $Spect^R$, P_{BAD}-*gam bet exo* P_{Tet}-*sg-ack*	Reisch et al., 2015
pSIJ8	*pSC101*, ori^{TS}, *araC*, Amp^R, P_{BAD}-*gam bet exo*	Jensen et al., 2015

Supplementary Table 2

Primers	Description	Primer Sequence	Source
CP1	Fwd.- Chloramphenicol cassette	GTCCAAGCGAGCTCGATATCAA	This study
CP2	Rev.- Chloramphenicol cassette_1	GTTGATCGGGCACGTAAGAGG	This study
CP3	Fwd.- Lpp	ATGAAAGCTACTAAACTGGTACTGGGCG	This study
CP4	Rev.- Lpp	TTACTTGCGGTATTTAGTAGCCATGTTGTC	This study
CP5	Fwd.- *Δlpp::cam* [w/ 50bp homology arm]	aatacttgtaacgctacatggagattaactcaatctagagggtattaataGTC CAAGCGAGCTCGATATCAA	This study
CP6	Rev.- *Δlpp::cam* [w/ 50bp homology arm]	acaaaaaaaatggcgcacaatgtgcgccatttttcacttcacaggtactaGT TGATCGGGCACGTAAGAGG	This study
CP7	Fwd.- Confirmatory PCR for *Δlpp::cam*	ACCCAGCGTTCGATGCTTCT	This study
CP8	Rev.- Confirmatory PCR for *Δlpp::cam*_1	AGCAGCCTGAACGTCGGAAC	This study
CP9	Rev.- Confirmatory PCR for *Δlpp::cam*_2	GCAGAATGGTGAACCAGAGCAA	This study
CP10	Rev.- Chloramphenicol cassette_2	GTGAATACCACGACGATTTCCG	This study
CP11	Fwd.- Fis [w/ 5'-NdeI restriction site]	AGAACATATGTTCGAACAACGCGTAAAT	This study
CP12	Rev.- Fis [w/5'-StyI restriction site]	ATAAACCTAGGTTAGTTCATGCCGTATTTTTTC A	This study
CP13	Fwd.- *Δfis::cam* [w/ 60bp homology arm]	aggcgttggaggcatacttcgaaaattttgcgtaaacagaaataaagagctga cagaactGTCCAAGCGAGCTCGATATCAA	This study
CP14	Rev.-*Δfis::cam* [w/ 60bp homology arm]	aaaaaggcgcttccccatgccgagtagcgcctttttaatcaagcatttagctaa cctgaaGTTGATCGGGCACGTAAGAGG	This study
CP15	Fwd.- Confirmatory PCR for *Δfis::cam*	TCCAAATGACCAGTTTCGGC	This study
CP16	Rev.- Confirmatory PCR for *Δfis::cam*	TTCACATCCTGTTCTCATGGTCAC	This study
CP17	Fwd.- *Δfis::spec* [w/ 60bp homology arm]	aggcgttggaggcatacttcgaaaattttgcgtaaacagaaataaagagctga cagaactTTATTTGCCGACTACCTTGGTGA	This study
CP18	Rev.- *Δfis::spec* [w/ 60bp homology arm]	aaaaaggcgcttccccatgccgagtagcgcctttttaatcaagcatttagctaa cctgaaATGCGCTCACGCAACTGGT	This study
CP19	Rev.- Spectinomycin cassette	CTTACCGTCGCGTTACTGTAAGAA	This study
CP20	Fwd.- *ΔcutC::tet* (w/ 60bp homology arm)	TACGAGCAAGCATCATATTGGGCGACATGATG CAACGGTAAAAATCATTTGGCCTGATGGCGTT CTTAAGACCCACTTTCACAT	This study

CP21	Rev.- *ΔcutC::tet* (w/ 60bp homology arm)	TCCGTGGTCCATTGAAGAGATCGTTGCCAGCG AGCAGTCGGCGTAATTAAGGAGTAAAAATGCT AAGCACTTGTCTCCTGTTTA	This study
CP22	Fwd.- Confimatory PCR for *ΔcutC::tet*	TCAACAGATCACGGTTATCGTTC	This study
CP23	Rev.- Confimatory PCR for *ΔcutC::tet*	ATGAGCGATTACCGAATCCGA	This study
CP24	Rev.- Confimatory PCR for TetA	TGCGATCTTTGTCGAACTATTCA	This study
